# Effects of Total Dissolved Gas Supersaturation on the Survival of Juvenile *Procypris rabaudi* and Juvenile *Myxocyprinus asiaticus* at Varying Water Depth in a Natural River

**DOI:** 10.3390/ani11113061

**Published:** 2021-10-26

**Authors:** Xiaoqing Liu, Wen Su, Chenyang Cao, Zhiqin Li, Yuanming Wang, Haoran Shi, Yao Yang, Liangfang Xu

**Affiliations:** 1 Key Laboratory of Fluid and Power Machinery, Ministry of Education, Xihua University, Chengdu 610039, China; 0120110023@mail.xhu.edu.cn (X.L.); 212020085900016@stu.xhu.edu.cn (W.S.); ccy269147022@163.com (C.C.); 0120010016@mail.xhu.edu.cn (H.S.); 0120080007@mail.xhu.edu.cn (Y.Y.); 0120020083@mail.xhu.edu.cn (L.X.); 2 Key Laboratory of Fluid Machinery and Engineering, Sichuan Province, Xihua University, Chengdu 610039, China; 3 School of Architecture and Civil Engineering, Xihua University, Chengdu 610039, China; 4 State Key Laboratory of Hydraulics and Mountain River Engineering, Sichuan University, Chengdu 610065, China

**Keywords:** total dissolved gas (TDG) supersaturation, *Procypris rabaudi*, *Myxocyprinus asiaticus*, survival probability, gas bubble disease (GBD)

## Abstract

**Simple Summary:**

Total dissolved gas (TDG) supersaturation severely threatens the survival of fish downstream of a dam in the Yangtze River due to flood discharge. However, most studies have been executed in the laboratory. Few works have evaluated the effects of TDG supersaturation on fish in natural rivers during periods of flood discharge. In the present study, we investigated the survival of two rare species (*Procypris rabaudi* and *Myxocyprinus asiaticus*) when subjected to TDG-supersaturated water at varied water depths in the natural river during periods of flood discharge. The results of this study showed that deeper water depths can increase the tolerance of juvenile *Procypris rabaudi* to TDG supersaturation in natural rivers during periods of flood discharge while it cannot improve the survival of juvenile *Myxocyprinus asiaticus*. Juvenile *Procypris rabaudi* were more vulnerable to TDG supersaturation than juvenile *Myxocyprinus asiaticus.* The study results can promote the protection of juvenile *Procypris rabaudi* and juvenile *Myxocyprinus asiaticus* (or other rare species) and contribute to the improvement of reservoir operation practices in the Yangtze River.

**Abstract:**

Total dissolved gas (TDG) supersaturation, which can be caused by flood discharge, results in gas bubble disease (GBD) in fish and threatens their survival downstream of dams. TDG supersaturation has become a serious environmental problem in the Yangtze River. Few studies have evaluated the effect of TDG supersaturation on fish in natural rivers during periods of flood discharge. To estimate fish tolerance to TDG supersaturation under natural conditions, juvenile *Myxocyprinus asiaticus* and juvenile *Procypris rabaudi* were exposed to TDG-supersaturated water for 96 h at various depths (0–0.3 m, 0.3–1.3 m, 1.3–2.3 m and 0–2.3 m) during periods of flood discharge of Dagangshan hydropower station. The results showed that juvenile *Procypris rabaudi* and juvenile *Myxocyprinus asiaticus* exhibited obvious GBD signs. An increase in exposure time decreased survival probability of the two species. Deeper water depths can increase the tolerance of juvenile *Procypris rabaudi* to TDG supersaturation in natural rivers during periods of flood discharge while it cannot improve the survival of juvenile *Myxocyprinus asiaticus*. Compared with juvenile *Myxocyprinus asiaticus*, juvenile *Procypris rabaudi* showed weaker tolerance of TDG supersaturation in shallow water, and juvenile *Procypris rabaudi* were more vulnerable to TDG supersaturation than juvenile *Myxocyprinus asiaticus* even if the TDG level (116%) was low.

## 1. Introduction

Dissolved gas supersaturation is a condition that results from natural processes (e.g., water cascades, changes in water temperature, photosynthesis) and human activity (flood discharge) [1,2,3,4,5]. The excessive dissolved gas in water results in gas bubble disease (GBD) in fish and threatens their survival [6,7,8]. The signs of GBD and its mortality rate in fish, such as *Esox lucius*, *Pomoxis nigromaculatus* and *Oncorhynchus tshawytscha*, have been reported due to dissolved gas supersaturation [9,10,11]. In the last ten years in China, a large number of high dams (>200 m) (e.g., Shuangjiangkou Dam, Xiluodu Dam, Xiangjiaba Dam and Lianghekou Dam) have been built or have begun construction on the Yangtze River. When floodwaters are released from these high dams, total dissolved gas (TDG) supersaturation occurs in the water downstream of the dams. TDG supersaturation maintains a high saturation level (130–143%) downstream of Three Gorges Dam and Ertan Dam during periods of flood discharge [5,12]. This may cause fatal effects in fish due to GBD. In 2014, the flood discharge at Xiluodu Hydropower Station caused the death of 40 tons of fish. Recently, many studies have been performed on the impact of TDG levels on the supersaturation tolerance of fish in the Yangtze River, for example, *Acipenser dabryanus, Procypris rabaudi*, *Hypophthalmichthys nobilis, Myxocyprinus asiaticus* and *Schizothorax prenanti* [13,14,15,16,17,18,19,20]. However, most of these studies have been executed in the laboratory. Few works have evaluated the effects of TDG supersaturation on fish in natural rivers during periods of flood discharge.

*Myxocyprinus asiaticus* and *Procypris rabaudi* inhabit in the upper Yangtze River and are endemic and rare species of Yangtze River. With the development of high dams in the Yangtze River, the populations of these two species have declined greatly owing to habitat destruction [21,22] and have been listed as key protected animals in China. In order to estimate their tolerance of supersaturation under natural conditions, it is necessary to determine the effect of water depth on their survival in TDG-supersaturated waters. Therefore, the aim of this study was to investigate the survival of these two species when subjected to TDG-supersaturated water at varied water depths in the natural river during periods of flood discharge. The study results can promote the protection of juvenile *Procypris rabaudi* and juvenile *Myxocyprinus asiaticus* (or other rare species) and contribute to the improvement of reservoir operation practices in the Yangtze River.

## 2. Materials and Methods

### 2.1. Ethics Statement

The experimental proposal was approved by Xihua University (202150-51). All experiments were executed in accordance with the animal management regulations of Sichuan Province in China.

### 2.2. Experimental Site and Experimental Fish

**Figure 1:**
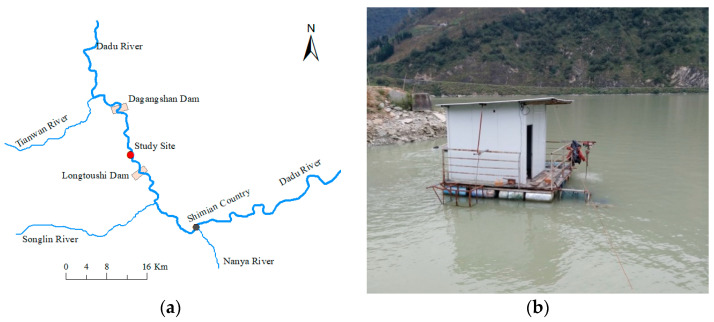
(**a**) Study site located 12 km downstream of Dagangshan Dam in the Dadu River; (**b**) Photograph of a test facility.

The experimental site (29°21′43.1″ N, 102°14′23.5″ E) is located in Shimian county (Sichuan province) and lies between Dagangshan Dam and Longtoushi Dam in the Dadu River (a tributary of the Yangtze River) (Figure 1). The test facilities were set up on 10 × 10 m covered barges that moored in the backwater region of the Dadu River approximately 12 km downstream from Dagangshan Dam.

In this experiment, juvenile *Myxocyprinus asiaticus* and juvenile *Procypris rabaudi* were obtained from the Sichuan Fisheries Research Institute (Chengdu, China) with authorization from the government. The weight and length of juvenile *Myxocyprinus asiaticus* were 16 ± 0.5 g and 17.6 ± 0.3 cm, respectively. The weight and length of juvenile *Procypris rabaudi* were 4.3 ± 0.4 g and 10.7 ± 1.1 cm, respectively.

### 2.3. Experimental Design

To investigate the survival of test fish exposed to TDG-supersaturated water at various depths, four different water depths (0–0.3 m, 0.3–1.3 m, 1.3–2.3 m and 0–2.3 m) were used for the tests (Table 1). Eight cages were used in the experiment. Two cages (length × width × height: 0.5 m × 0.5 m × 0.5 m) were placed at water depths from 0–0.3 m. Two cages of the same size were placed at the 0.3–1.3 m depths. Two cages of the same size were placed at the 1.3–2.3 m depths. Two cages (length × width × height: 0.5 m × 0.6 m × 2.3 m) were placed at water depths from 0–2.3 m. Furthermore, the control group (TDG 100%) was established in a tank (720 L) filled with aerated river water in the river bank. Two replicates were also set up for the control group.

Before the experiment, the tested fish were held in aerated river water (dissolved oxygen (DO) level: 5.81–8.02 mg L^−1^; water temperature: 16–17 °C; TDG level: 98–100%) for 24 h to allow them to acclimate to the new environment. After acclimation, 20 juvenile *Myxocyprinus asiaticus* and 20 juvenile *Procypris rabaudi* were placed into each cage (Table 1). The same number of test fish was placed in the control group. During the experiment, the tested fish were observed at 8.00 a.m. in the morning and at 1:00 and 7:00 p.m. in the afternoon by raising the cages to the surface. The signs of GBD and the number of dead fish were recorded. The TDG level was continually measured by a Point Four Tracker (Pentair Aquatic Eco-Systems Inc., Coquitlam, Canada). A multiparameter water quality sonde (6600, YSI Inc., Yellow Springs, OH, USA) was used to measure the pH value, temperature and DO level. The weight and length of dead fish were measured with an electronic scale and a ruler. The whole experiment lasted for 96 h from 4 to 8 July 2017.

### 2.4. Data Analysis

In this study, the survival process of juvenile *Procypris rabaudi* and juvenile *Myxocyprinus asiaticus* in the TDG supersaturated water with varied depth was investigated with the survival probability. The survival probability is calculated based on the following mathematical formula:

(1) $$p=\left(1-\frac{n}{N}\right)\times 100\%$$

where p, n, and N are the survival probability, number of deaths and total number of the test fish in each case, respectively.

Furthermore, the survival analysis of the test fish was performed with an accelerated failure time (AFT) model in R version 3.6.1 [23,24]. The application of the AFT model to detect the effect of TDG supersaturation on fish has been described in our previous studies [20,21,25]. Briefly, the AFT model used in this study is depicted as follows:

(2) $$T=\exp(\beta \cdot d)\cdot T_{0}$$

where T is the time-to-death of juvenile *Myxocyprinus asiaticus* (or juvenile *Procypris rabaudi*) experienced the exposure of TDG-supersaturated water at various depths, and $T_{0}$ stands for the time-to-death of these two species in the control group. The water depth of 0–0.3 m was considered the control group in the analysis. The variable d represents the experimental water depth, and the unknown coefficient β represents the influence of the water depth on the time-to-death of juvenile *Myxocyprinus asiaticus* (or juvenile *Procypris rabaudi*) exposed to TDG supersaturation. Hypothetically, $T_{0}$ should have a loglogistic distribution, and the water depth was treated as the categorical variable to fit the survival data. The parameters involved in the AFT model were evaluated by the maximum likelihood method. Thus, the survival S(t) can be determined at a given time (t) by using the following equation:

(3) $$S(t)={\left(1+{\left(t\cdot \exp{\left(\varepsilon \right)}^{-1}\cdot \exp{\left(\beta \cdot d\right)}^{-1}\right)}^{1/\phi }\right)}^{-1}$$

where the unknown parameters ε (intercept) and φ (scale) can be determined in the AFT model. Furthermore, the survival curve determined from the AFT model can completely describe the survival process of the test fish. The *survfit* function was applied to determine the estimated coefficients and their standard errors from the survival curve related to each experimental condition. In addition, the function *survdiff* was selected to analyze and compare the survival curves among the experimental groups. The Holm method was used to examine the *p*-value correlation [26]. The average survival time (AST) calculated from the AFT model was used to estimate the effect of water depth on the survival of the test fish experienced the TDG exposure by one-way analysis of variance (ANOVA). The least significant difference test was used for a post hoc multiple comparison with the one-way ANOVA. Tamhane’s T2 test was applied when homogeneity of variance was not confirmed. The significance level was set at p < 0.05.

## 3. Results

### 3.1. TDG Supersaturation Analysis and GBD Signs

**Figure 2:**
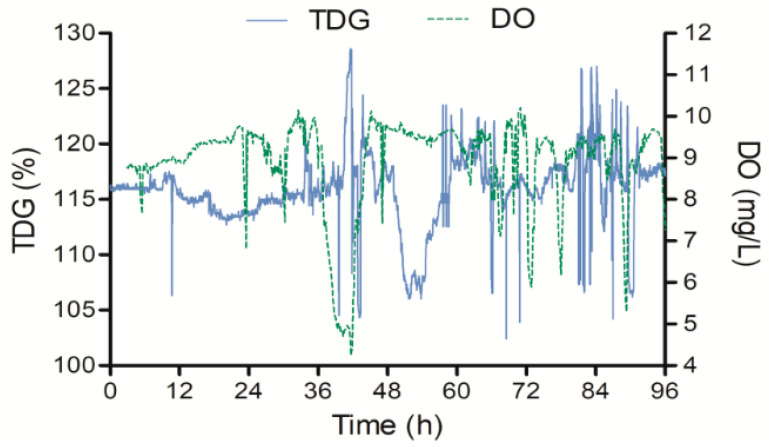
The values of total dissolved gas (TDG) and dissolved oxygen (DO) in the 96-h observation period.

During the observation period, the water temperature was 14.9–17.4 °C. The TDG and DO values are shown in Figure 2. The TDG level varied around 115% during the first 36 h. A high value (127% TDG) and a low level (105% TDG) appeared in 36–48 h. The range of the TDG level was 105–115% in 48–60 h, and the TDG level fluctuated obviously at approximately 84 h. The average values of TDG and DO were 116% and 8.89 mg L^−1^, respectively. During the experiment, the test fish showed obvious GBD signs, such as gas bubbles in their fins and gills.

### 3.2. Survival of the Test Fish under TDG Supersaturation at Various Water Depths

**Figure 3:**
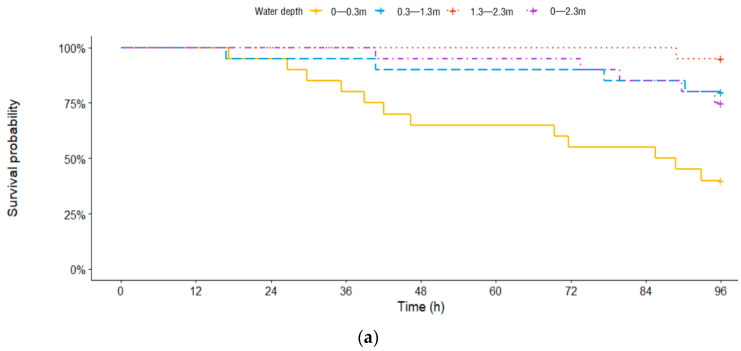

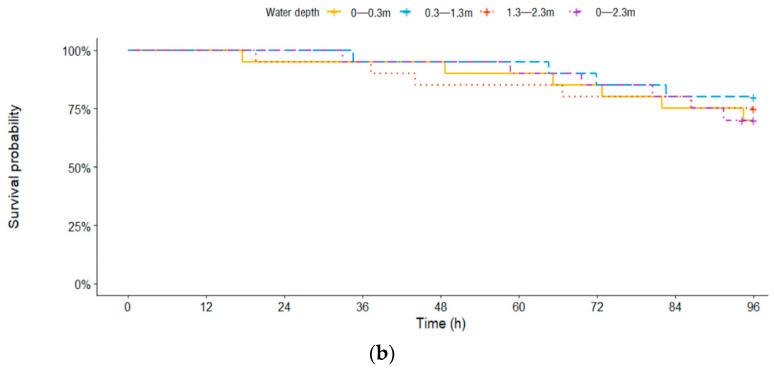
Survival curves of the test fish under total dissolved gas (TDG) supersaturation (116%) at different water depths (0–0.3 m, 0.3–1.3 m, 1.3–2.3 m and 0–2.3 m). (**a**) *Procypris rabaudi*; (**b**) *Myxocyprinus asiaticus*.

The survival probability of the test fish increased with increased water depth while increased TDG supersaturation exposure time led to decreased survival probability (Figure 3). Figure 3a shows that there was a 95% survival probability for juvenile *Procypris rabaudi* in the 12–24 h period at depths of 0–0.3 m and 0.3–1.3 m. After exposure for 48 h, 65% of juvenile *Procypris rabaudi* survived at 0–0.3 m, and no test fish died at 1.3–2.3 m. At the end of 96 h of exposure, the survival probability of juvenile *Procypris rabaudi* had decreased to 40%, 80%, 95% and 75% at the 0–0.3 m, 0.3–1.3 m, 1.3–2.3 m and 0–2.3 m depths, respectively. Additionally, as shown in Figure 3b, the survival probability of juvenile *Myxocyprinus asiaticus* was 95% in the 12–24 h period at 0–0.3 m and 1.3–2.3 m, respectively. No dead juvenile *Myxocyprinus asiaticus* appeared at 0.3–1.3 m or 0–2.3 m during the same exposure period. After exposure for 48 h, the survival probability of juvenile *Myxocyprinus asiaticus* decreased to 85% at 1.3–2.3 m, while there was a 95% survival probability at other water depths (0–0.3 m, 0.3–1.3 m and 0–2.3 m). Until the exposure time of 96 h, the survival probability of juvenile *Myxocyprinus asiaticus* was 70%, 80%, 75% and 65% at 0–0.3 m, 0.3–1.3 m, 1.3–2.3 m and 0–2.3 m, respectively. Throughout the experiment, all juvenile *Procypris rabaudi* and *Myxocyprinus asiaticus* survived at 100% TDG.

Table 2 lists the estimated parameters obtained from the AFT model fitting. The effect of water depth on the survival of juvenile *Procypris rabaudi* and *Myxocyprinus asiaticus* was estimated using the absolute values of the coefficients. The results showed that water depth had a strong effect on the survival of juvenile *Procypris rabaudi* exposed to TDG supersaturation. Compared with shallow water (0–0.3 m), deep water significantly increased the survival of juvenile *Procypris rabaudi* (|Coeff.${\;\beta }_{0.3-1.3\;m}$| = 0.63, p = 0.05 and |Coeff.${\;\beta }_{1.3\;-\;2.3\;m}$| = 1.18, p < 0.01). However, there was no significant difference in the survival of juvenile *Procypris rabaudi* between 0–0.3 m and 0–2.3 m (Coeff.$\;\beta_{0\;-\;2.3\;m}$| = 0.58, p=0.06  > 0.05). Furthermore, as shown in Table 2, no significant difference in juvenile *Myxocyprinus asiaticus* survival was found between the water depth of 0–0.3 m and the other water depths (|*Coeff.*$\;\beta_{0.3\;-\;1.3\;m}$| = 0.48, p = 0.23 > 0.05, |*Coeff.*$\;\beta_{1.3\;-\;2.3\;m}$| = 0.26, p=0.32 > 0.05, and |*Coeff.*$\;\beta_{0-2.3\;m}$| = 0.19, p=0.6 > 0.05).

**Figure 4:**
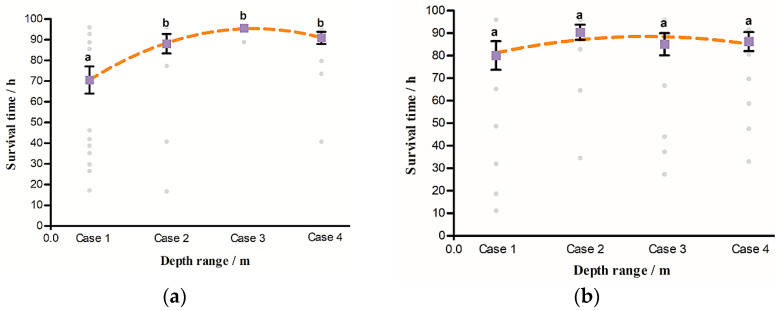
The survival time of test fish exposed to total dissolved gas (TDG) supersaturation at different water depths (Case 1: 0–0.3 m; Case 2: 0.3–1.3 m; Case 3: 1.3–2.3 m; Case 4: 0–2.3 m). (**a**) *Procypris rabaudi*; (**b**) *Myxocyprinus asiaticus*. Each value is showed in terms of the mean ± S.E., *n* = 40. Blue dots represent the average survival time (AST) of the test fish at each water depth. Grey dots represent the survival time of each test fish. The orange line represents the best-fit regression model. Mean values are significantly different if they are not marked with the same lowercase letter (p < 0.05).

In this study, the average survival time (AST) was also used to assess the survival of juvenile *Procypris rabaudi* and juvenile *Myxocyprinus asiaticus* experienced the exposure of TDG-supersaturated water at varied water depths (Figure 4 and Table 3). As shown in Figure 4a, the AST was significantly lower at water depths of 0–0.3 m than at other water depths (0.3–1.3 m: F = 4.67; df = 1,40; p=0.03  < 0.05; 1.3–2.3 m: F = 14.54; df = 1,40; p < 0.01; 0–2.3 m: F = 7.99; df = 1,40; p  < 0.01). There were no significant differences in the AST of *Procypris rabaudi* at water depths of 0.3–1.3 m, 1.3–2.3 m and 0–2.3 m. For juvenile *Myxocyprinus asiaticus* (Figure 4b), no significant differences were observed in the AST values at 0.3–1.3 m, 1.3–2.3 m and 0–2.3 m compared with that at 0–0.3 m (0.3–1.3 m: F = 0.31; df = 1,40; p=0.58  > 0.05; 1.3–2.3 m: F = 0.04; df = 1,40; p=0.83 > 0.05; 0–2.3 m: F = 0.10; df = 1,40; p=0.75 > 0.05).

In addition, the results in Table 3 show that the AST values of juvenile *Procypris rabaudi* were 72.61 h, 85.85 h, 92.88 h and 89.36 h at 0–0.3 m, 0.3–1.3 m, 1.3–2.3 m and 0–2.3 m, respectively. The AST values of juvenile *Myxocyprinus asiaticus* were 80.01 h, 90.36 h, 84.72 h and 86.26 h in the same water depths, respectively. The AST value of juvenile *Procypris rabaudi* was lower than that of juvenile *Myxocyprinus asiaticus* at 0–0.3 m and 0.3–1.3 m. However, juvenile *Procypris rabaudi* had higher AST values than juvenile *Myxocyprinus asiaticus* at 1.3–2.3 m and 0–2.3 m.

## 4. Discussion

In the mid-1960s, it gradually became evident that a serious dissolved gas problem existed in the Columbia River system [7]. Westgard (1964) observed adult *Oncorhynchus tshawytscha* suffering gas bubble disease at the McNary Spawning Channel [9]. Subsequently, the effect of gas supersaturated water on *Oncorhynchus tshawytscha, Oncorhynchus* and *Salmo gairdneri* were investigated in the Nechako River and the Columbia River [2,11]. In China, some studies have been carried to estimate the effect of TDG on endemic species of Yangtze River (e.g., *Acipenser dabryanus, Schizothorax prenanti* and *Procypris rabaudi*) in the laboratory [17,18,19]. However, relatively few studies have investigated the effect of TDG supersaturation on fish in natural rivers. Xue et al. [27] indicated that all juvenile *Ctenopharyngodon idella* died at 0–1 m water depth (average TDG: 125%; exposure time: 140 h) when the Xiangjiaba Dam discharged floodwaters in the upper Yangtze River in 2014. At depths of 1–2 m, the survival rate of the species was approximately 60%. In the next year, they also found that approximately 30% *Procypris rabaudi* survived at 0–0.5 m water depth in 100 h (average TDG: 123%; average temperature: 22.3 °C). Cao et al. [28] indicated that no juvenile *Myxocyprinus asiaticus* survived at 0–0.7m water depth in 60 h (average TDG: 123%; average temperature: 22.3 °C) downstream of Xiangjiaba Dam in August 2014 and September 2015, but the mortality decreased with increasing water depth. In our results, the survival probability of juvenile *Procypris rabaudi* and juvenile *Myxocyprinus asiaticus* was 40% and 70% at 0–0.3 m water depth (average TDG: 116%; exposure time: 96 h; average temperature: 16.1 °C), respectively. This may indicate that increasing TDG level has great effects on the survival of these species in natural rivers. However, the effect of temperature should not be ignored because increasing water temperature can decrease the survival of fish exposed to TDG based on Yuan et al. [29]. They found that an increase in water temperature (from 12 to 20 °C) decreased median survival time (from 18.62 to 5.22 h) of juvenile *Schizothorax prenanti* exposed to 130% TDG for 96 h.

Furthermore, in the study of Yuan et al. [30], 23% and 43% of adult *Schizothorax prenanti* survived at water depths of 0–1 m and 1–2 m in TDG-supersaturated water (average TDG: 117%), respectively. At the same water depths, adult *Schizothorax davidi* showed a higher survival rate (53%) after 6 d of exposure. Their results also showed that the survival rates of juvenile *Schizothorax prenanti*, juvenile *Schizothorax davidi* and juvenile *Leptobotia eloungata* were 86%, 70% and 96% at water depth of 0–1 m, respectively. The juveniles exhibited greater tolerance than the adults under the same conditions. Juvenile *Leptobotia elongata* showed the highest tolerance of all fish in their study. In the present study, the survival probability of juvenile *Procypris rabaudi* was 40%, 80% and 95% at 0–0.3 m, 0.3–1.3 m and 1.3–2.3 m, respectively, while that of juvenile *Myxocyprinus asiaticus* was 70%, 80% and 75% at the same water depths (Figure 3). Compared with the surface water (0–0.3 m), deeper depths increased the survival of the test fish. This was consistent with the studies of Xue et al. [27], Cao et al. [28] and Yuan et al. [30]. Furthermore, the water depth made a greater contribution to the survival of juvenile *Procypris rabaudi* than to the survival of juvenile *Myxocyprinus asiaticus*. Juvenile *Myxocyprinus asiaticus* showed a higher tolerance to TDG supersaturation in shallow water (0–0.3 m) than juvenile *Procypris rabaudi*. When the water depth was 0–2.3 m, the survival probability of juvenile *Procypris rabaudi* rose from 40% (0–0.3 m) to 75%. We speculate that juvenile *Procypris rabaudi* can escape from TDG supersaturation by swimming to deeper depths. Cao et al. [31] found that *Myxocyprinus asiaticus* can avoid highly TDG-supersaturated water (140%–150% TDG). At present, no observations have been made to investigate TDG supersaturation avoidance by *Myxocyprinus asiaticus* at other TDG levels (<140%). In our results, there was only a small change in the survival probability of juvenile *Myxocyprinus asiaticus* between 0–0.3 m and 0–2.3 m (from 70% to 65%) after 96 h of exposure. We speculate that juvenile *Myxocyprinus asiaticus* did not exhibit obvious avoidance ability at a low TDG level (116%).

It has been shown that GBD in fish can lead to fish death owing to hypoxemia, which is caused by embolism in the blood vessels [25,32,33]. In the present study, juvenile *Procypris rabaudi* and juvenile *Myxocyprinus asiaticus* subjected to TDG-supersaturated water at different water depths exhibited signs of GBD, such as bubbles in their gills and fins (caudal fin, ventral fin and dorsal fin). In addition, our results also demonstrated that increased water depth extended the survival time of the test fish and increased their survival. The possible explanation for this finding is that the compensation depth of water decreased the development of GBD [34]. Previous research demonstrated that higher TDG level (>125%) led to high mortality (nearly 100%) in fish after 96 h of exposure [13,19,22,31]. Relatively high survival probabilities for juvenile *Procypris rabaudi* and juvenile *Myxocyprinus asiaticus* were observed in the study. This may be related to the low TDG level (116%) in addition to the effect of compensation depth. In addition, one possible reason for this finding is that the exposure time of 96 h at the low TDG level was not long enough to cause high mortality in the fish [28,35,36].

Based on the results of the AFT model (Table 2 and Figure 4), there were significant differences in the survival of juvenile *Procypris rabaudi* between the surface water (0–0.3 m) and the other water depths (0.3–1.3 m,1.3–2.3 m and 0–2.3 m). No significant differences were observed for juvenile *Myxocyprinus asiaticus* survival under the same conditions because of high survival probability. This indicated that water depth has a greater effect on the survival of juvenile *Procypris rabaudi* than on that of juvenile *Myxocyprinus asiaticus* exposed to TDG supersaturation (116% TDG). Additionally, our results also showed that the AST of juvenile *Procypris rabaudi* was lower at 0–0.3 m and 0.3–1.3 m than that of juvenile *Myxocyprinus asiaticus* (Table 3). In comparison to juvenile *Myxocyprinus asiaticus*, juvenile *Procypris rabaudi* exhibited a weaker tolerance to TDG supersaturation in the relatively shallow water layer, and juvenile *Procypris rabaudi* was more vulnerable to TDG supersaturation even if the TDG level was low. However, when the test fish were provided with more space (0–2.3 m) to swim freely, the AST of juvenile *Procypris rabaudi* was higher than that of juvenile *Myxocyprinus asiaticus*. The reasons for this result may be that juvenile *Procypris rabaudi* can exhibit their ability to avoid TDG.

According to the results of a previous laboratory study, the mortality of juvenile *Procypris rabaudi* (weight: 2.41–2.48 g; length: 6.6–7.2 cm; water depth: 0.2 m; temperature: 25 °C) was minimal in TDG-supersaturated water (115% TDG) after 60 h of exposure [13]. No juvenile *Myxocyprinus asiaticus* (average weight: 39.54 g; average length: 11.54 cm; water depth: 0.2–0.5 m; temperature: 21 °C) died at 120% TDG after exposure for 48 h [15]. In our study, the survival probabilities of juvenile *Procypris rabaudi* (average weight: 4.3 g; average length: 10.7 cm) and juvenile *Myxocyprinus asiaticus* (average weight: 6 g, average length: 7.6 cm) were 40% and 70% respectively, at 0–0.3 m depth in the Dadu River (average temperature: 16.1 °C) after 96 h of TDG supersaturation exposure. This may be related to the peak TDG levels that occurred during the test (Figure 2). In addition, existing results showed that suspended sediment might accelerate fish death in TDG-supersaturated water [19,21,22]. For example, Li et al. [22] found that an increase in suspended sediment concentration (from 200 to 1000 mg L^−1^) increased mortality (from 10 to 70%) of juvenile *Myxocyprinus asiaticus* exposed to 125% TDG for 4 h, while increasing suspended sediment concentration (from 200 to 1000 mg L^−1^) caused 20 to 81% mortality in juvenile *Procypris rabaudi* exposed to 125% TDG for 10 h [21]. The water temperature and the size of the fish affect fish tolerance to TDG supersaturation [20,29,33,37,38]. The differences in TDG supersaturation tolerance between our results and the results of previous laboratory studies might be explained by the above factors. To provide accurate predictions of what will really happen, the results of laboratory and field bioassay experiments must be interpreted in terms of all discernible natural conditions. Thus, due to the lack of field results in China, more field bioassay experiments should be performed on the effects of TDG supersaturation in fish, such as examining the avoidance ability of different species (especially rare fish), examining lethal and sub-lethal effects for all life stages of same species and examining effects caused by repeated exposure in natural rivers during the discharge of flood. Furthermore, although some useful results were obtained in the present study, we only set up two replicates for each water depth. The results may have limitations in assessing the effect of TDG supersaturation on the survival of juvenile *Procypris rabaudi* and juvenile *Myxocyprinus asiaticus* at varying water depth.

## 5. Conclusions

Our study indicates that deeper water depths can increase the tolerance of juvenile *Procypris rabaudi* to TDG supersaturation in natural rivers during periods of flood discharge while it cannot improve the survival of juvenile *Myxocyprinus asiaticus*. Compared with juvenile *Myxocyprinus asiaticus*, juvenile *Procypris rabaudi* show a weaker tolerance to TDG supersaturation in shallow water even if the TDG level is low. According to our results, we speculate that juvenile *Procypris rabaudi* may have an ability to avoid TDG supersaturation (average value: 116%), but that juvenile *Myxocyprinus asiaticus* did not show this ability. In this study, 40–95% juvenile *Procypris rabaudi* and *Myxocyprinus asiaticus* can survived at 116% TDG for 96 h at 0–0.3 m, 0.3–1.3 m and 1.3–2.3 m depths. If the exposure time is sufficiently long or the TDG level is higher (>120%), the survival of juvenile *Procypris rabaudi* and juvenile *Myxocyprinus asiaticus* may be seriously threatened even at water depths of 2.3 m. Maintaining a low TDG level (<116%) and deep water depths (>3 m) downstream of dams is necessary for the protection of *Procypris rabaudi* and *Myxocyprinus asiaticus* (or other rare species). In addition, we find that fluctuating TDG levels with a mean of 116% TDG cause greater mortality in both species compared to static exposures in the lab. To provide an effective measurement for managing GBD effects in fish, more field studies should be performed. The thresholds of effect of TDG should be determined in future research for the formulation of reservoir operation systems during the flood season. Moreover, a long exposure time to high TDG levels should be avoided. Therefore, some measures should be taken to eliminate and reduce harm to fish from TDG supersaturation, for example, shortening discharge periods, increasing water depth and restricting TDG levels.

## Figures and Tables

**Table 1:** Experimental conditions.

Species	Groups	Number of Test Fish at Various Depths			
		0–0.3 m (Case 1)	0.3–1.3 m (Case 2)	1.3–2.3 m (Case 3)	0–2.3 m (Case 4)
*Myxocyprinus asiaticus*	Group 1	20	20	20	20
	Group 2	20	20	20	20
*Procypris rabaudi*	Group 1	20	20	20	20
	Group 2	20	20	20	20

**Table 2:** Estimated parameters for the accelerated failure time (AFT) model of the influence of water depth on the survival of juvenile *Procypris rabaudi* and juvenile *Myxocyprinus asiaticus* exposed to total dissolved gas (TDG) supersaturation.

Scheme.	Factors	Parameters	Estimated Coefficients (Coeff.)		
			Coeff. (S.E.) ^a^ Walid-Z Pr(>|Z|) ^b^		
*Procypris rabaudi*	Intercept	μ	4.45 (0.20)	22.05	<0.01 **
	Depth	β	0.58 (0.31)_0–2.3 m_	1.89	0.06
			0.63(0.33)_0.3–1.3 m_	1.94	0.05 *
			1.18(0.44)_1.3–2.3 m_	2.66	<0.01 **
	Log (scale)	σ	−0.73 (0.18)	−3.98	<0.01 **
*Myxocyprinus asiaticus*	Depth	β	0.19 (0.36)_0–2.3 m_	0.52	0.60
			0.48(0.42)_0.3–1.3 m_	1.19	0.23
			0.26(0.42)_1.3–2.3 m_	0.98	0.32
	Log (scale)	σ	−0.57 (0.20)	−2.82	<0.01 **

^a^ Water depths were deemed categorical variables for the survival data in the accelerated failure time (AFT) model fitting. ^b^ Asterisks represent significance (*: *p* < 0.05; **: *p* < 0.01); S.E.: standard error.

**Table 3:** The average survival time (AST) of juvenile *Procypris rabaudi* and juvenile *Myxocyprinus asiaticus* in the total dissolved gas (TDG)-supersaturated water at different water depths (mean ± S.E., *n* = 20).

Water Depth (m)	Species	Average Survival Time (h)
0–0.3	*Procypris rabaudi* *Myxocyprinus asiaticus*	72.61 ± 6.56
		80.01 ± 6.40
0.3–1.3	*Procypris rabaudi* *Myxocyprinus asiaticus*	85.85 ± 5.46
		90.36 ± 3.37
1.3–2.3	*Procypris rabaudi* *Myxocyprinus asiaticus*	92.88 ± 2.70
		84.72 ± 5.21
0–2.3	*Procypris rabaudi* *Myxocyprinus asiaticus*	89.36 ± 3.16
		86.26 ± 4.21

## Data Availability

All data generated or analyzed during this study are available from the corresponding authors upon reasonable request.
